# The BET inhibitor OTX015 reactivates latent HIV-1 through P-TEFb

**DOI:** 10.1038/srep24100

**Published:** 2016-04-12

**Authors:** Panpan Lu, Xiying Qu, Yinzhong Shen, Zhengtao Jiang, Pengfei Wang, Hanxian Zeng, Haiyan Ji, Junxiao Deng, Xinyi Yang, Xian Li, Hongzhou Lu, Huanzhang Zhu

**Affiliations:** 1 State Key Laboratory of Genetic Engineering, and Key Laboratory of Medical Molecular Virology of Ministry of Education/Health, School of Life Sciences, Fudan University, Shanghai, China; 2 Department of Infectious Diseases, and Key Laboratory of Medical Molecular Virology of Ministry of Education/Health, Shanghai Public Health Clinical Center, Fudan University, Shanghai, China

## Abstract

None of the currently used anti-HIV-1 agents can effectively eliminate latent HIV-1 reservoirs, which is a major hurdle to a complete cure for AIDS. We report here that a novel oral BET inhibitor OTX015, a thienotriazolodiazepine compound that has entered phase Ib clinical development for advanced hematologic malignancies, can effectively reactivate HIV-1 in different latency models with an EC_50_ value 1.95–4.34 times lower than JQ1, a known BET inhibitor that can reactivate HIV-1 latency. We also found that OTX015 was more potent when used in combination with prostratin. More importantly, OTX015 treatment induced HIV-1 full-length transcripts and viral outgrowth in resting CD4^+^ T cells from infected individuals receiving suppressive antiretroviral therapy (ART), while exerting minimal toxicity and effects on T cell activation. Finally, biochemical analysis showed that OTX015-mediated activation of HIV-1 involved an increase in CDK9 occupancy and RNAP II C-terminal domain (CTD) phosphorylation. Our results suggest that the BET inhibitor OTX015 may be a candidate for anti-HIV-1-latency therapies.

Human immunodeficiency virus type 1 (HIV-1) is an incurable infection that causes acquired immune deficiency syndrome (AIDS). Antiretroviral therapy (ART) suppresses HIV-1 to undetectable levels and partially restores immune function in infected individuals. However, interrupting ART causes the virus to rapidly rebound to pretreatment levels^1^,^2^. The main cause of treatment failure is due to the existence of latent HIV-1 reservoirs. Resting CD4^+^ T cells harboring integrated and transcriptionally silent proviruses are the best-characterized reservoir, can evade host immune surveillance and resume production of infectious viral particles once the therapy is interrupted^3^,^4^,^5^. This latent reservoir, likely established within days of infection^6^, persists throughout life due to its extreme stability, makes life-long ART necessary and represents the primary hurdle to an HIV-1 cure^7^.

Understanding the molecular mechanisms of HIV-1 latency is a prerequisite for designing new treatments that aim to eliminate the reservoirs. Much progress has recently been made to elucidate the molecular mechanisms underlying HIV-1 proviral latency^8^,^9^,^10^, mostly acting at the level of transcriptional suppression of the viral promoter long terminal repeats (LTR). Transcriptional blocks to productive HIV-1 replication include epigenetic modifications at the HIV-1 LTR^11^,^12^, the presence of transcriptional repressors and inadequate availability of activation-dependent transcription factors, such as human positive transcription elongation factor b (P-TEFb), an essential co-factor for Tat, etc^13^,^14^.

Several therapeutic strategies have been proposed to eliminate or control the pool of latent HIV-1. These involve either complete elimination of all persistent HIV-1, called sterilizing cure, or immunological control of persistent HIV-1, called functional cure^15^. The “shock and kill” strategy has gained much attention as a basis for sterilizing cure^16^ and finding efficient small molecule latency reversing agents (LRAs) to induce virus production without causing global T cell activation has been a research priority in the HIV-1 field in recent years^17^,^18^. To this end, several small molecules have been shown to stimulate HIV-1 transcription in latently infected cells^18^. However, these compounds are toxic, mutagenic or ineffective in trials involving enlarged sample size and prolonged treatment^10^,^19^,^20^,^21^,^22^. Thus, more effective and specific latency activators are urgently needed.

Recently, the therapeutic potential of pharmacologic inhibition of members of the bromodomain and extraterminal domain (BET) family has received much attention. The BET protein family is a well-conserved class of transcriptional regulators that are distinguished by the presence of tandem bromodomains, conserved domains and an extraterminal domain^23^,^24^. It is thought that targeting the binding of BET proteins to chromatin may provide an effective way to regulate HIV-1 gene expression, and in particular, transcription elongation^25^,^26^. Notably, a number of studies have reported that the BET inhibitor JQ1 can reactivate HIV-1 in different latency models and also in ART treated patients when combined with an HDACi or PKC agonist^25^,^27^,^28^,^29^,^30^. Recently, a novel oral inhibitor of BRD2/3/4, the thienotriazolodiazepine compound OTX015, suitable for human use, has received much attention^31^,^32^ and entered phase Ib clinical trials for advanced hematologic malignancies (NCT01713582)^33^. Here, we examined the impact of OTX015 on HIV-1 latency. Our data indicate that OTX015 can effectively reactivate latent HIV-1 through an increase in cyclin-dependent kinase 9 (CDK9) occupancy and RNAP II CTD phosphorylation in HIV-1 latency models *in vitro.* Furthermore, this effect is potently enhanced by combining OTX015 with prostratin. Importantly, this treatment also induced latent HIV-1 expression in primary CD4^+^ T cells *ex vivo* from individuals with suppressive ART, while exerting minimal toxicity and detrimental effects on T cell activation.

## Results

### OTX015 induces HIV-1 expression in latently infected cell lines *in vitro*

**Figure 1:**
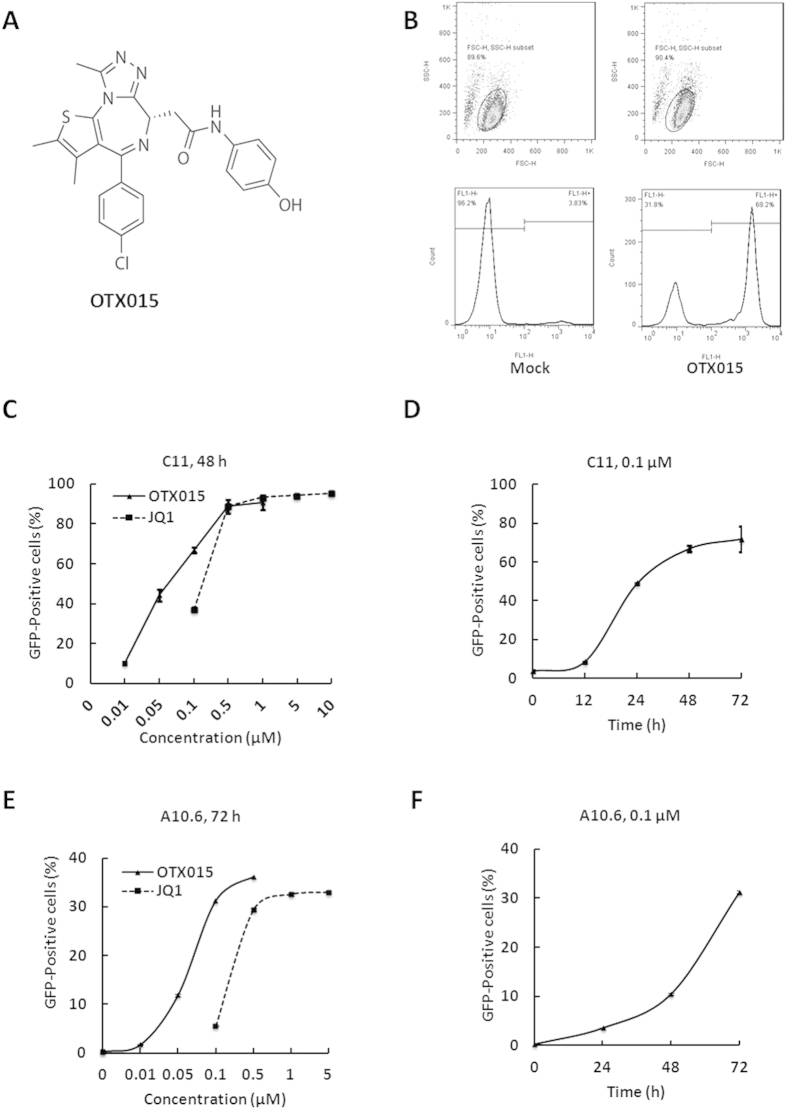
OTX015 induces HIV-1 expression *in vitro* in latent HIV-1 cell culture models. (**A**) The structure of OTX015. (**B**) J-Lat clone C11 cells were treated with 0.1 μM OTX015 for 48 h and induction of GFP, representing the level of HIV-1 transcription, was measured by flow cytometry and presented as fluorescence histograms. (**C**) C11 cells were treated with OTX015 or JQ1 for 48 h at the indicated concentrations. Results are expressed as a percentage of GFP-positive cells within the entire population (**D**) C11 cells were treated with 0.1 μM OTX015 for the indicated time period, and the results are expressed as percentage of GFP-positive cells in the entire population. (**E**,**F**) A10.6 cells were treated and analyzed as in (**C,D**).

The structure of OTX015 is shown in Fig. 1A. In order to determine the potential of OTX015 to induce HIV-1 expression, we used the C11 cell line that had been previously constructed in our lab^34^. This cell line is a clonal latently infected Jurkat T-cell line with a single provirus integrated into intron of RNPS1 and a green florescence protein (GFP) gene under the control of HIV-1 LTR. After treatment with 0.1 μM OTX015 for 48 h, the percentage of GFP-positive cells, representing HIV-1 LTR-driven GFP expression, was found to be 68% higher than the cells subjected to mock treatment (Fig. 1B). Moreover, OTX015 was found to stimulate HIV-1 LTR transcription in C11 cells in a dose- and time-dependent manner (Fig. 1C,D). As shown in Fig. 1C, when the concentration of OTX015 increased from 0.01 μM to 1 μM, the percentage of GFP-positive cells rose from 10.2% to 90.8%. To further evaluate the efficiency of OTX015, we calculated the 50% effective concentration (EC_50_) on C11 cells. Compared to another BET inhibitor JQ1, which is known to reactivate HIV from latency^25^,^30^, the EC_50_ value of OTX015 was 0.05845 μM, which is 1.95 times lower than that of JQ1 (0.1142 μM). As shown in Fig. 1D, after C11 cells were treated with 0.1 μM OTX015, the percentage of GFP-positive cells increased as a function of time. Seventy-two hours after treatment, we observed that the percentage of GFP-positive cells was as high as 71.8%.

To examine whether similar results could be obtained in other latently infected T cells, we used J-Lat clone A10.6 cells, which is also a Jurkat T cell line latently infected by HIV-1^35^. The results from these cells indicated that OTX015 can activate latent HIV-1 transcription in a dose- and time-dependent fashion. Furthermore, the EC_50_ value of OTX015 was 0.06378 μM, 4.34 times lower than that of JQ1 (0.2769 μM) (Fig. 1E,F). Taken together, these data show that OTX015 is highly potent in reactivating latent HIV-1 in different latently infected Jurkat T cell models.

### The effect of OTX015 on reactivation of latent HIV-1 is potently enhanced in combination with prostratin

**Figure 2:**
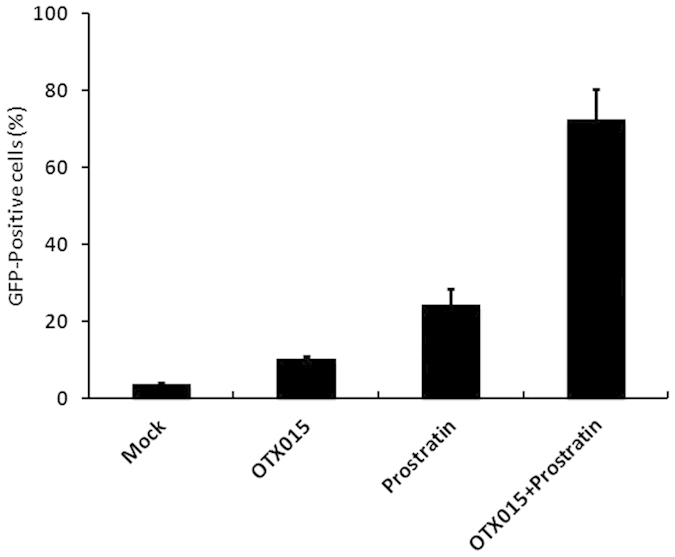
The effect of OTX015 on reactivation of latent HIV-1 is potently enhanced in combination with prostratin. C11 cells were mock-treated or treated with either OTX015 (0.01 μM), prostratin (0.2 μM), or OTX015 (0.01 μM)/prostratin (0.2 μM). The effect of activation of the HIV-1 promoter was determined by quantifying GFP-positive cells 48 hours after treatment using flow cytometry. A summary of the activation assays is presented as a series of histograms.

Several molecular pathways are involved in the establishment and maintenance of HIV-1 latency^8^,^9^,^10^ and in order to optimally reactivate latent HIV-1 expression, we utilized the PKC agonist prostratin in combination with OTX015. C11 cells were treated with OTX015 (0.01 μM), prostratin (200 nM) or OTX015 (0.01 μM)/prostratin (200 nM) for 48 h. A lower concentration was used in these assays since OTX015 was found to be very potent in reactivating latent HIV-1 expression (Fig. 1) and the combined effect of OXT015 with other LRAs would be difficult to distinguish at higher concentrations. As shown in Fig. 2, in the absence of stimulation, only 3.6% of C11 cells expressed GFP. The stimulation of C11 cells with either OTX015 or prostratin alone induced GFP expression in only 10.2% and 24.2% of cells, respectively. When cells were co-treated with OTX015 and prostratin, we observed robust activation of virus production with 72.4% of C11 cells becoming GFP-positive.

To assess whether the activity of the combined drugs meets the criteria for drug synergy, we compared the experimentally observed effect of the combined drugs to the effect predicted under the Bliss independence model^36^. This model assumes that if two compounds act through different mechanisms, their effects are merely additive in the absence of synergistic interactions. In contrast, the effects of drug combinations that are greater or lesser than the ideal Bliss independence prediction imply synergy or antagonism, respectively. We observed that combination of OTX015 and prostratin resulted in 68.8% of cells being GFP positive (over and above the DMSO control), which was much higher than the predicted 25.8% of cells, calculated according to Materials and methods description. This result demonstrates that OTX015 and prostratin had synergistic effects on the reactivation of HIV-1 from latency in a C11 cell line.

### OTX015 induces latent HIV-1 expression in primary CD4^+^ T cells from individuals with suppressive ART *ex vivo*

**Figure 3:**
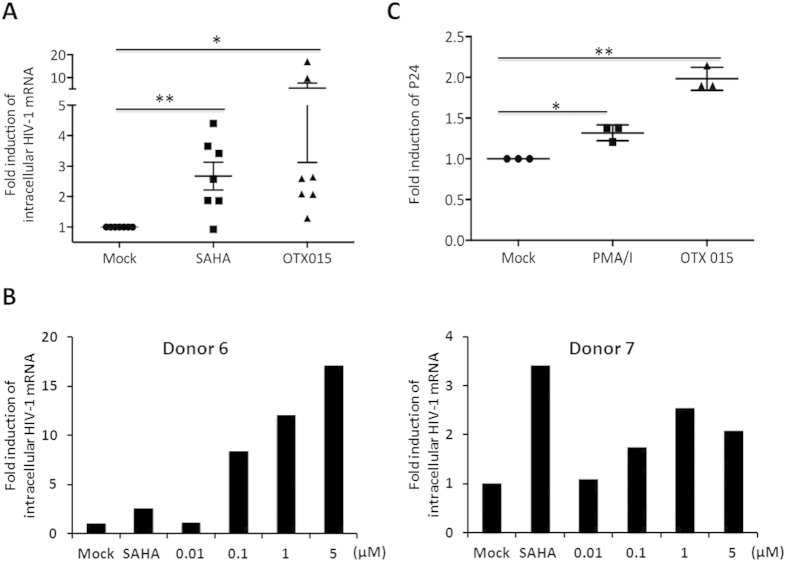
OTX015 induces latent HIV-1 expression in *ex vivo* primary CD4^+^ T cells from individuals with suppressive ART. (**A**) Resting CD4^+^ T cells were obtained from seven infected individuals and treated *ex vivo* with either 5 μM OTX015 or 500 nM SAHA for 18 h. Intracellular HIV-1 mRNA levels were measured using RT-qPCR for the Poly A region and presented as fold induction relative to the DMSO control. (**B**) Resting CD4^+^ T cells from two of the seven infected individuals in A were treated with the indicated concentrations of OTX015 or 500 nM SAHA for 18 h and then examined as in A. (**C**) Resting CD4^+^ T cells were obtained from three infected individuals and treated *ex vivo* with either OTX015 (5 μM) or PMA (50 ng/ml)/ionomycin (1 μM) for 18 h and then co-cultured with MOLT-4/CCR5 for determination of viral outgrowth. HIV-1 expression was detected by enzyme-linked immunosorbent assay (ELISA) for the p24 antigen at 14d and presented as fold induction relative to the DMSO control. *p < 0.05, **p < 0.01.

We examined the ability of OTX015 to induce latent HIV-1 expression in resting CD4^+^ T cells. Resting CD4^+^ T cells purified from the peripheral blood of HIV-1 infected individuals on suppressive ART were incubated with 5 μM OTX015 or suberoylanilide hydroxamic acid (SAHA) for 18 h. HIV-1 mRNA expression levels were measured by RT-qPCR with primers/probe specific for the HIV-1 3′ polyadenylation (poly A) region, which can verify that latent HIV-1 produced full-length transcripts^37^. The fold induction of HIV-1 RNA transcription differed somewhat between donors due to the inherently variable response of latently infected resting CD4^+^ T cells to LRAs. Despite this, OTX015 induced an increase in HIV-1 transcription in all the seven donors, among which six donors had >2 fold increase. However, after treatment with SAHA, HIV-1 transcription was also observed in six out of seven donors, although only four donors showed >2 fold increase and the remaining two donors showed a 1.9 fold increase (Fig. 3A). To further evaluate the capacity of low concentration of OTX015 to reactivate latent HIV-1, we choose resting CD4^+^ T cells isolated from two of the seven donors above and treated them with a broad titration of the drug (0.01–5 μM). Results showed that 0.1 μM and 1 μM OTX015 also can increase HIV-1 mRNA expression, but a higher dose of drug was expected to induce more potent reactivation of latent HIV-1 from patient samples (Fig. 3B).

We next asked whether OTX015 treatment induced viral outgrowth. To address this question, purified resting CD4^+^ T cells from infected individuals on ART were treated with OTX015 for 18 h. These cells were subsequently cultured for 14 d with a transformed CD4^+^ T cell line (MOLT-4/CCR5) that supports robust HIV-1 replication but does not induce allogeneic stimulation of resting CD4^+^ T cells, to permit viral outgrowth^37^. Viral outgrowth was assessed by an ELISA assay for HIV-1 p24 antigen in the culture supernatant^38^. Phorbol 12-myristate 13-acetate plus ionomycin (PMA/I) treated cultures served as a positive control. In the three donors participated, OTX015 induced 1.9–2.1 fold viral outgrowth from cells compared to the untreated control, whereas PMA/I-treated cultures only showed 1.2–1.4 fold increase (Fig. 3C). These results suggest that OTX015 is effective in reactivating HIV-1 from latently infected cells *ex vivo*.

### OTX015 displays minimal toxicity in primary CD4^+^ T cells

**Figure 4:**
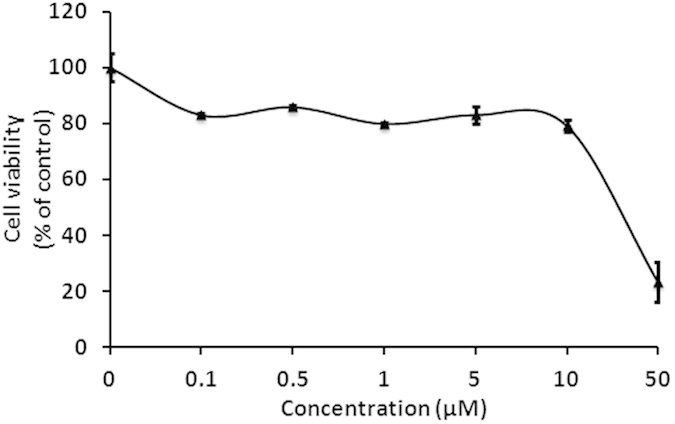
OTX015 displays minimal impact on cell viability in PBMCs at its active concentration. PBMCs from HIV-negative donors were treated with OTX015 at the indicated concentrations for 48 h and then cell viability was measured by the CCK-8 method. Changes in OD450 between different drug concentrations indicated the percentage of cell viability.

To be clinically applicable, effective LRAs should be highly potent, minimally cytotoxic and able to penetrate anatomical sanctuaries and immune cell types without inducing global T cell activation^39^. Therefore, we sought to determine the cytotoxicity of OTX015 and examine its effect on T cell activation. To test OTX015 cytotoxicity, PBMCs of healthy HIV-negative donors were treated with differing concentrations of OTX015 for 48 h and subjected to CCK-8 assays. We did not find a clear reduction in cell viability when the concentration increased from 0.1 μM to 10 μM, however, cell viability was strongly reduced at concentrations >10 μM (Fig. 4). We calculated the 50% cytotoxic concentration (CC_50_) of OTX015 on PBMCs and found it to be 28.75 μM (Fig. 4), much higher than the EC_50_ value on C11 cells (0.05845 μM) (Fig. 1C) or on A10.6 cells (0.06378 μM) (Fig. 1E). The therapeutic index (TI) of OTX015 is more than 400, indicating that it is very safe at its active concentration.

**Figure 5:**
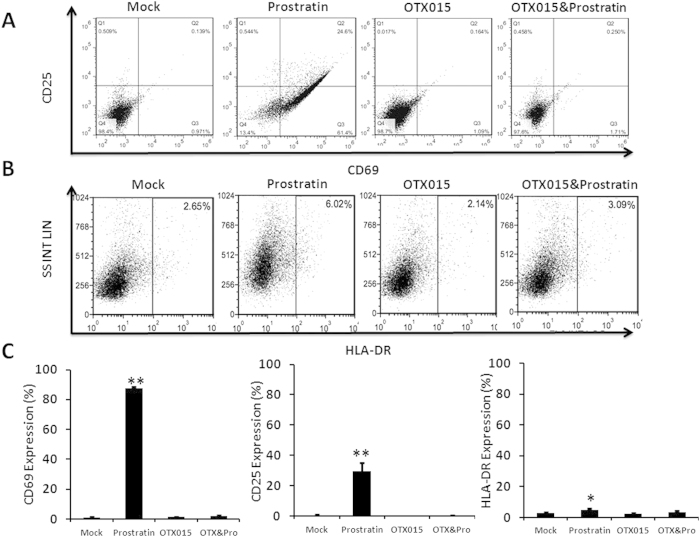
OTX015 does not induce expression of T cell activation markers on primary CD4^+^ T cells. (**A**,**B**) CD4^+^ T cells were isolated from the peripheral blood of healthy HIV-negative donors and treated with prostratin (1 μM), OTX015 (5 μM) or OTX015 (0.01 μM)/prostratin (0.2 μM) for 48 h and the expression of the cell activation markers CD25, CD69 and HLA-DR was detected by flow cytometry using specific antibodies. (**C**) Summary of effects of OTX015 and prostratin on CD25, CD69 and HLA-DR expression are presented as histograms. *p < 0.05, **p < 0.01.

The major disadvantage of current therapeutic agents is their propensity to non-specifically activate bystander T cells^40^. To address this problem, we examined CD4^+^ T cells purified from PBMCs of healthy HIV-negative donors for the expression of T cell activation biomarkers following 48 h of stimulation with OTX015. Flow cytometric analysis showed that OTX015 treatment did not cause any significant change in the expression of the T cell markers CD25, CD69 and HLA-DR. However, consistent with previously published results^41^, there robustly increased CD25 and CD69 expression was observed after treatment with prostratin (Fig. 5). Aforementioned observations have shown that OTX015 and prostratin had synergistic activation (Fig. 2), we wanted to know whether they can reduce the effects on T cell activation when combined as a lower concentration was used in these assays. Results showed that combination treatment do not induce expression of CD25, CD69 and HLA-DR (Fig. 5).

**Figure 6:**
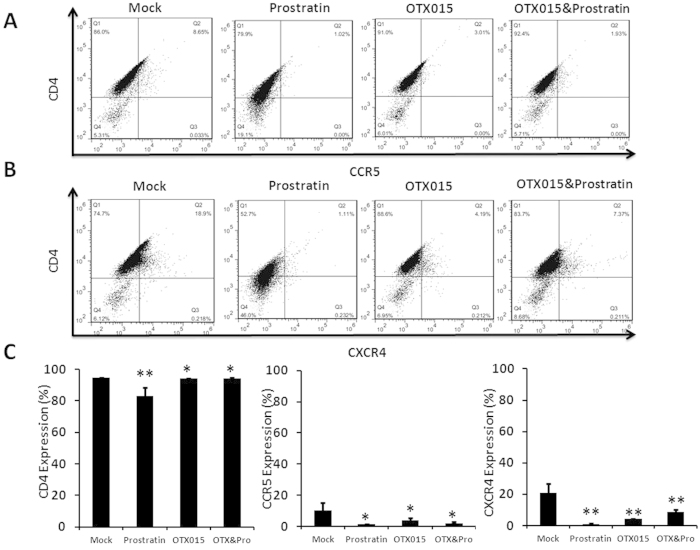
OTX015 does not induce expression of HIV-1 receptors on primary CD4^+^ T cells. (**A**,**B**) CD4^+^ T cells were isolated from the peripheral blood of healthy HIV-negative donors and treated with prostratin (1 μM), OTX015 (5 μM) or OTX015 (0.01 μM)/prostratin (0.2 μM) for 48 h and the expression of CD4, CCR5 and CXCR4 was detected by flow cytometry using specific antibodies. (**C**) Summary of effects of OTX015 and prostratin on CD4, CCR5 and CXCR4 expression are presented as histograms. *p < 0.05, **p < 0.01.

Cell surface expression of HIV-1 receptors/co-receptors is important for viral attachment and entry into immune cells. Recent studies have shown that prostratin and analogs disrupt HIV-1 receptor/co-receptor expression, which may have protective effects against HIV-1 infection^42^,^43^. Conversely, SAHA was reported to increase susceptibility of naive CD4^+^ T cells to HIV-1 acquisition^44^. We sought to examine the effect of OTX015 on the expression of cell surface HIV-1 receptors and co-receptors in CD4^+^ T cells. Primary CD4^+^ T cells from the peripheral blood of healthy HIV-negative donors were treated with OTX015 for 48 h and evaluated for the expression of CD4, CCR5 and CXCR4 using flow cytometry. Our data showed that OTX015 treatment did not cause an increase in the expression of these HIV receptors/co-receptors (Fig. 6), suggesting that OTX015 may not pose the risk of increasing the susceptibility of CD4^+^ T cells to HIV-1 infection during the reactivation of HIV-1 latency. We also examined the influence of combination treatment of OTX015 and prostratin on HIV-1 receptors and found that they do not increase the expression of CD4, CCR5 and CXCR4, even slightly reduce them, although not remarkably as high concentration of prostratin (Fig. 6).

In summary, at the active concentration of OTX015, no significant impact was seen on cell viability and neither T cell activation biomarkers nor HIV-1 receptors/co-receptors were induced, suggesting that OTX015 may be a potential LRA candidate for further evaluation *in vivo*.

### OTX015 predominantly stimulates Tat-dependent HIV-1 transcription

**Figure 7:**
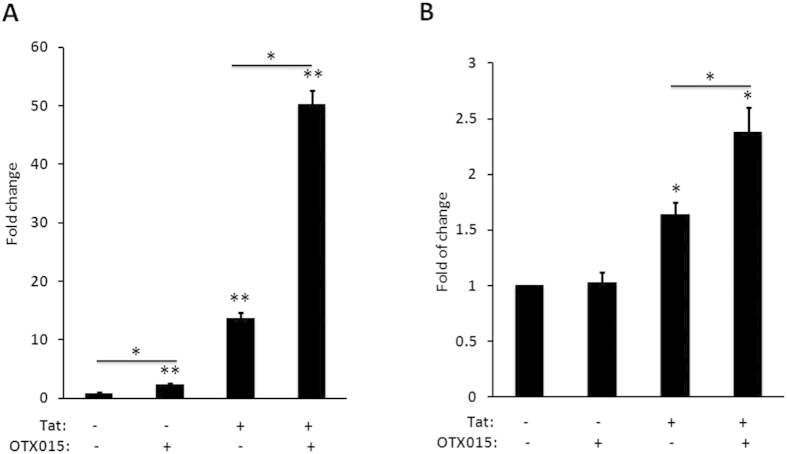
OTX015 stimulates predominantly Tat-dependent HIV-1 transcription. (**A**) HeLa-based TZMbl cells containing an integrated HIV LTR-luciferase construct were nucleofected with a plasmid expressing Tat (+) or a negative control plasmid (−). Cells were then treated with OTX015 (0.5 μM) or DMSO (−) as indicated. Whole cell extracts were assayed for luciferase activity. (**B**) Primary CD4^+^ T cells, isolated from the peripheral blood of healthy HIV-negative donors, were nucleofected with plasmid expressing Tat or a negative control plasmid at the presence of LTR-luciferase construct. Cells were then treated and examined as in A. *p < 0.05, **p < 0.01.

HIV-1 encoded Tat protein recruits the cellular pause relief factor, P-TEFb, to the transactivation response element (TAR) which is an RNA stem loop formed at the 5′ extremity of all viral transcripts, thereby enhancing processive RNA Polymerase II (RNAP II) transcription. Fluctuation in Tat levels below a critical threshold has been proposed to be important in the establishment of latency in CD4^+^ T cells^45^. Given the critical role for Tat in overcoming HIV-1 latency^27^,^46^,^47^,^48^,^49^, we wanted to determine if the stimulatory effect of OTX015 is also related to Tat. To this end, we examined the effect of OTX015 on the expression of an integrated luciferase reporter gene driven by the HIV-1 LTR in the Hela-based TZMbl cell line with or without Tat. OTX015 alone (0.5 μM) only induced 2.5-fold LTR-driven luciferase expression relative to the mock control. However, when transfected with a plasmid expressing Tat, activation of HIV-1 LTR by OTX015 was dramatically increased up to a maximum of 50.3-fold. We then asked whether Tat expression alone would be sufficient to bypass the requirement for OTX015 to reverse HIV-1 latency. The data showed that mere expression of Tat only induced luciferase expression 13.8-fold, much lower than the levels seen when Tat and OTX015 were added together (Fig. 7A).

Ruling out a cell type-specific phenomenon, the ability of OTX015 to activate the Tat-dependent HIV-1 transcription was also demonstrated in primary CD4^+^ T cells from healthy donors (Fig. 7B). Together, these results indicate that the mere expression of Tat alone was largely insufficient to overcome latency, but that Tat does play a critical role in OTX015-mediated activation of the latent HIV-1 LTR.

### OTX015 increases CDK9 T-loop phosphorylation

**Figure 8:**
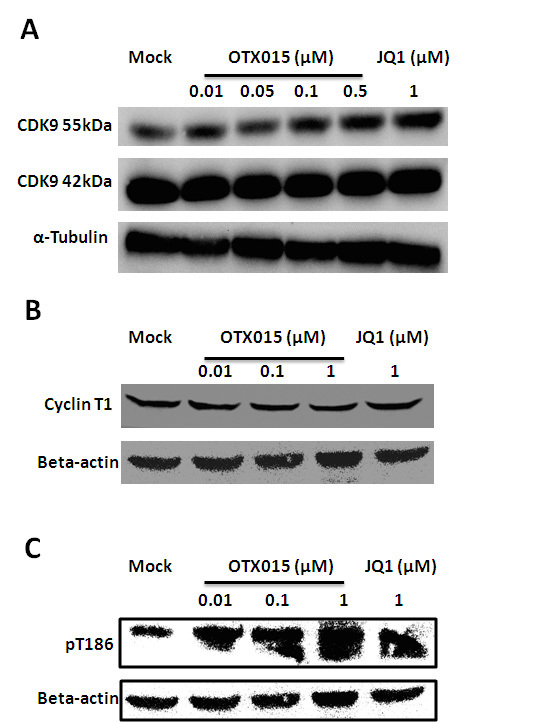
OTX015 increases CDK9 T-loop phosphorylation in Jurkat C11 cells. After Jurkat C11 cells were treated with different concentrations of OTX015 or JQ1 as indicated for 24 h, total CDK9 (**A**), Cyclin T1 (**B**) levels and the extent of CDK9 phosphorylation on Thr186 (pT186) (**C**) were determined by Western Blotting.

P-TEFb, a kinase composed of the catalytic subunit CDK9 and a regulatory subunit Cyclin T1, increases RNAP II activity by hyper-phosphorylating the CTD of RNAP II^13^,^50^. As transcription elongation from the HIV-1 promoter uniquely depends on P-TEFb^51^, we determined if the effect of OTX015 on latent cells can influence P-TEFb. To our surprise, in Jurkat C11 cells treated with a broad titration of OTX015, neither CDK9 nor Cyclin T1 was found to increase when detected with specific antibodies (Fig. 8A,B). We then asked whether increased phosphorylation of Thr186, which is located at the tip of the CDK9 T-loop and required for CDK9′s catalytic activity^52^,^53^, could be detected with help from OTX015. Indeed, the CDK9 phosphorylation was found to increase (Fig. 8C). Increased CDK9 phosphorylation was also found in JQ1 treated cells (the positive control), which is consistent with previously published results^25^.

### OTX015 increases P-TEFb recruitment to the HIV-1 LTR and induces RNAP II CTD phosphorylation

**Figure 9:**
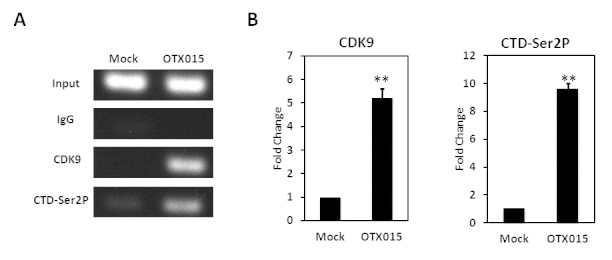
OTX015 increases CDK9 recruitment to the HIV-1 LTR and induces RNAP II CTD phosphorylation. (**A**) C11 cells were mock-treated or stimulated with OTX015 (0.5 μM) for 6 h. ChIP assays were performed using antibodies against CDK9, Pol II CTD-Ser2P or normal mouse IgG. PCR primers specific for the LTR promoter were used to amplify the DNA isolated from the immunoprecipitated chromatin as described in Materials and methods. (**B**) Each ChIP experiment was repeated three times to confirm reproducibility of the results. Real-time quantitation of the fold change relative to the DMSO control is shown. **p < 0.01.

P-TEFb functions during the elongation phase of transcription by phosphorylating the CTD of RNAP II, thus overcoming a block to transcriptional elongation^54^,^55^. In light of the aforementioned observation that OTX015 stimulated P-TEFb activity through CDK9 phosphorylation, we wanted to further determine whether OTX015 affected the occupancy of P-TEFb at the HIV-1 promoter and the phosphorylation of the RNAP II CTD. To this end, quantitative ChIP analysis of the binding of these factors to a small region overlapping the HIV-1 transcriptional start site was performed in Jurkat C11 cells before and after OTX015 treatment. As expected, OTX015 induced a noticeable increase (5.2-fold) in the level of CDK9 bound to the HIV-1 promoter compared with the DMSO control. This, in turn, resulted in enhanced phosphorylation (9.6-fold increase over the control) of the RNAP II CTD on Ser2 (CTD-Ser2P) (Fig. 9). These results indicate that OTX015 activates latent HIV-1 through increasing P-TEFb recruitment to the LTR and then inducing RNAP II CTD phosphorylation and viral transcription.

In addition, these findings provide some evidence that the combination of OTX015 and prostratin synergistically activated HIV-1 LTR-driven GFP expression in C11 cells (Fig. 2). This activation is likely due to their cooperative activation of different stages of the HIV-1 transcription cycle—prostratin is known to activate NF-κB and ELL2, which in turn promote RNAP II recruitment and entry into the pause site, thus working on transcription initiation^25^,^56^,^57^, whereas OTX015 acts subsequently to enhance Pol II elongation.

## Discussion

Latent reservoirs of HIV-1 are the main barrier to the eradication of infection. The “shock and kill” strategy is the main focus of current research efforts. In this strategy, LRAs would be used to induce the reactivation of latent HIV-1 in the presence of ART. After reactivation, latent HIV-1 expression would induce viral cytopathic effects, immune clearance, and cell death, thereby purging latently infected cells while uninfected cells are protected by ART^15^,^16^. The first challenge to this strategy is to find ways to efficiently reactivate latent HIV-1. A number of agents have been explored^40^ and some, such as Disulfiram^58^ (NCT01286259), Romidepsin^59^,^60^ (NCT01933594) (NCT02092116), Panobinostat^61^ (NCT01680094), Vorinostat^62^,^63^ (NCT01319383) (NCT01365065), have entered into clinical trials^10^. To our disappointment, no significant reduction in the size of the latent HIV-1 reservoir has been seen in any of these studies.

Promoter-proximal pausing of initiated RNAP II on integrated HIV-1 proviral DNA has long been recognized as a major rate-limiting step in viral gene expression^14^,^55^. To overcome this restriction, the viral protein Tat interacts directly with TAR and recruits P-TEFb, which is composed of CDK9 and Cyclin T1 domains, to the HIV-1 LTR. Enzymatically active CDK9, which requires phosphorylation of Thr186 in its T-loop, phosphorylates the CTD of RNAP II and the negative elongation factors, thereby increasing the rate of HIV-1 transcription^13^. The BET proteins also obstruct HIV-1 gene expression in this signaling pathway. For example, BRD4 has been shown to compete with Tat for P-TEFb binding, inhibiting the activation of transcription elongation^25^. BRD2 also acts as an endogenous negative regulator of HIV-1 latency^30^. Therefore, BET inhibitors can indirectly lead to transcriptional effects by altering the available cellular pool of P-TEFb and may provide a novel therapeutic approach to reverse HIV-1 latency^64^,^65^.

In the current study, we evaluated the anti-latency ability of a novel oral BET inhibitor OTX015. Our data showed that OTX015 can effectively reactivate HIV-1 in different Jurkat cells-based latency models and, notably, its EC_50_ value was 1.95–4.34 times lower than JQ1. However, while the EC50 value was similar in both cell lines, the magnitude of latency reversal was significantly different; a maximum GFP positive population of 90% was achieved with C11 cells, but a maximum of 36% of A10.6 cells became GFP positive. This is possibly because that the pseudovirus used in establishment *in vitro* HIV-1 latency model are different. The genome of J-Lat A10.6 clone does not contain the integration of a complete HIV-1 genome but the vector pEV731 that is a minimum transcription-competent HIV-1 provirus expressing the viral Tat protein together with GFP upon LTR promoter activation^35^. The genome of C11 clone contain the vector pNL4-3 that is a HIV-1 constructor imported EGFP-encoding gene in a nef open reading frame mutated into Env and Vpr genes^34^. Besides, the HIV-1 integration sites in different latently infected cells are also different and it influences the virus transcription^9^. As mentioned above, we need to specify that these data came from *in vitro* studies in cell lines latently expressing defective provirus with potentially complete ability to initiate replication. Further investigations to confirm this using primary latency model are warranted.

Combination therapy can reduce the chance of evolving drug resistance, decrease side effects and achieve enhanced potency^66^. As such, we also examined whether OTX015 could synergize with other activators. Our results showed that its effect is potently enhanced in combination with prostratin. Moreover, they do not induce global T cell activation as prostratin treatment alone. Also this provided several clues for exploring the mechanism of OTX015 in reversing HIV-1 latency. Further experiments are needed to explore the correlation between OTX015 and other types of activators.

Remarkably in our study, we found OTX015 treatment alone could induce latent HIV-1 full-length transcripts and viral production in resting CD4^+^ T cells from infected individuals on suppressive ART. Importantly, unlike other LRAs, such as PHA, PMA and prostratin, which effectively reverse HIV-1 latency *ex vivo* but induce global T cell activation^67^ and are too toxic for clinical use, OTX015 exerted no effects on T cell activation and its CC_50_ was much higher than its EC_50_. But importantly to point out that the increases in viral outgrowth induced by OTX015 were very modest, we need to optimize the concentration and timepoint of OTX015, as well as its combination treatment with other activators, in resting CD4^+^ T cells from HIV-1 infected patients to further improve its efficacy in inducing HIV-1 expression in the future. Nevertheless, these existing data support further investigation of OTX015 as a potential candidate in HIV-1 cure studies.

It has often been debated whether or not the effect of BET inhibitors on latent HIV-1 depends on Tat. Zhu *et al.* reported modest stimulation of an HIV-LTR reporter by JQ1 in the absence of Tat. However, a combination of JQ1 and Tat resulted in more cells with reactivated viruses^29^. Li *et al.* showed that the effect of JQ1 in activating latent HIV-1 is Tat-dependent in Jurkat 1G5 and HeLa-based NH1 and NH2 cells^25^. On the other hand, Boehm *et al.* showed that this activation is P-TEFb dependent, but independent from the viral Tat protein^30^. Our studies performed in Hela-based TZMbl cells and primary CD4^+^ T cells isolated from healthy donors demonstrate that Tat plays a critical role in OTX015-mediated activation of latent HIV-1.

Levels of active P-TEFb are pivotal in promoting the switch to productive elongation, and therefore play a large role in controlling the expression of viral genes^68^. Our data show that OTX015-mediated activation of HIV-1 involved an increase in CDK9 T-loop phosphorylation, which is required for the catalytic activity of CDK9. Through ChIP assays, we further found that OTX015 stimulation promoted CDK9 recruitment directly to the HIV-1 LTR, and induced RNAP II CTD phosphorylation and viral transcription. As BET proteins are similar in structure^69^, it is unclear which target is closely related to OTX015-mediated reversal of HIV-1 latency. However, our studies yield important mechanistic insights into the mechanism of action of BET inhibitors.

In summary, we have provided strong evidence that the BET inhibitor OTX is a potent antagonist of HIV-1 latency, and acts by increasing the occupancy of CDK9 at the HIV-1 LTR and inducing RNAP II CTD phosphorylation. We also demonstrated the important role of the viral protein Tat in regulating OTX-mediated HIV-1 LTR gene expression. However, it is important to extend these observations to a wider population of latent cells from infected individuals undergoing ART to confirm the potential of OTX015 as a drug candidate in anti-HIV-1-latency therapy.

## Methods

### Cell culture

Jurkat C11 cells^34^ (constructed in our lab) or A10.6 cells^35^,^70^ (obtained from NIH AIDS Reagent Program) were cultured in RPMI1640 medium with 10% fetal bovine serum (FBS) and 1% Pen/strep in a 37 °C incubator containing 5% CO_2_. Reactivation of HIV-1 LTR was performed by treating cells with OTX015 (Selleckchem), JQ1 (Selleckchem) or prostratin (Sigma). HIV-1 reactivation was quantified by GFP expression using flow cytometry (Caliber, BD) and analyzed using FlowJo Software.

### Quantitative analysis of latency-reversing agent combinations

We used the Bliss independence model as a metric to evaluate the latency-reversing activity of drug combinations^36^. This model is defined by the equation


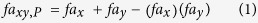


where *fa*_*xy*,*P*_ is the predicted fraction affected by a combination of drug *x* and drug *y*, given the experimentally observed fraction affected by treatment with drug *x* (*fa*_*x*_) or drug *y* (*fa*_*y*_) individually. The experimentally observed fraction affected by a combination of drug *x* and drug *y* (*fa*_*xy,O*_) can be compared with the predicted fraction affected, which is computed using the Bliss model (*fa*_*xy,P*_) as follows:


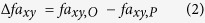


If Δ*fa*_*xy*_ > 0 with statistical significance, then the combined effect of the two drugs exceeds that predicted by the Bliss model and the drug combination displays synergy. If Δ*fa*_*xy*_ = 0, then the drug combination follows the Bliss model for independent action. If Δ*fa*_*xy*_ < 0 with statistical significance, then the combined effect of the two drugs is less than that predicted by the Bliss model and the drug combination displays antagonism. In our analysis, the fraction affected was calculated as follows for the percentage of GFP-positive cells: *fa*_*x*_ = % GFP-positive cells after treatment with drug *x*–% GFP-positive cells treated with the DMSO control.

### Isolation of resting CD4^+^ T cells

Shanghai Public Health Clinical Center approved this study, and the methods were carried out in accordance with the guidelines of Bullen, C. K. *et al.*^37^. All research participants in this study gave written informed consent. HIV-1-infected individuals were enrolled under the criteria of suppression of viremia to undetectable levels (<50 copies/ml) on ART for at least six months. Peripheral blood mononuclear cells (PBMCs) were purified using density gradient centrifugation from whole blood. CD4^+^ T lymphocytes were enriched by negative depletion (CD4^+^ T cell Isolation Kit, Miltenyi Biotec). Resting CD4^+^ T lymphocytes were further enriched by depletion of CD69^+^, CD25^+^ or human leukocyte antigen DR^+^ (HLA-DR^+^) cells. The purity of the isolated resting CD4^+^ lymphocytes was verified by flow cytometry and was typically greater than 95%.

### Measurement of intracellular HIV-1 RNA transcripts

Purified resting CD4^+^ T cells (5 × 10^6^) were treated with SAHA or OTX015 for 18 h in the presence of 10 μM T20 and collected for RNA purification. Total RNA was extracted using the ZR-96 Viral RNA Kit (Zymo Research). cDNA synthesis was performed using the GoScript Reverse Transcription System containing an oligo(dT)_15_ primer (Promega). Real-time PCR was performed in triplicate using the QuantiFast SYBR Green PCR Kit (QIAGEN) on a Roche LightCycler 480 II machine. Primers and probes specific for the HIV-1 3′ polyadenylation (poly A) region were designed as described^37^,^71^: forward (5′-3′) CAGATGCTGCATATAAGCAGCTG (9501–9523), reverse (5′-3′) TTTTTTTTTTTTTTTTTTTTTTTTGAAGCAC (9629-poly A) and probe (5′-3′) FAM-CCTGTACTGGGTCTCTCTGG-MGB (9531–9550). Results from the triplicate samples for each drug treatment were averaged and presented as fold change relative to DMSO control.

### MOLT-4/CCR5 outgrowth assay

Purified resting CD4^+^ T cells (5 × 10^6^) were treated with PMA plus ionomycin or OTX015 for 18 h and washed with 1 ml sterile PBS to remove residual drug. Resting CD4^+^ T cells were then cultured with MOLT-4/CCR5 cells in 8 ml RPMI1640 medium plus 10% FBS in individual wells in six-well culture plates. After four days and seven days of culture, wells were resuspended and split 1:2 with the medium volume adjusted to 8 ml per well. After 14 days of culture, viral outgrowth was assessed using the HIV-1 p24 Antigen ELISA kit (ZeptoMetrix)^37^.

### Measurement of cell viability and detection of T cell activation markers and HIV-1 receptors/co-receptors

PBMCs from healthy individuals were placed in 96-well plates and incubated with OTX015 for 48 h. Cell viability was measured using the Cell Counting Kit-8 (Dojindo Molecular Technologies) as described^72^. To measure changes in the cell activation status and presence of HIV-1 receptors/co-receptors, CD4^+^ lymphocytes isolated from healthy donors were incubated with prostratin, OTX015 or OTX015/prostratin for 48 h and immunostained with anti-CD25, anti-CD69, anti-HLA-DR, anti-CD4, anti-CCR5 or anti-CXCR4 antibodies (BD-Biosciences) for 20 min at 4 °C. Cells were fixed in 1% PFA and analyzed by flow cytometry.

### Transient transfection and luciferase assays

TZMbl cells^73^, grown in Dulbecco’s modified Eagle’s medium (DMEM) supplemented with 10% FBS, were plated at 1 × 10^5^ cells/well in 24-well culture plates 24 h before transfection and then transfected with Tat or pcDNA 3.1 plasmid using Lipofectamine 2000 (Invitrogen) following the manufacturer’s instructions. Primary CD4^+^ T cells (5 × 10^6^) isolated from healthy donors were transfected with Tat or pcDNA 3.1 plasmid at the presence of LTR-Luc construct using Amaxa^®^ Human T Cell Nucleofector^®^ Kit (Lonza) following the manufacturer’s instructions and then plated in 6-well culture plates. At 24 h post-transfection, the cells were mock-treated or treated with OTX015. At 48 h post-treatment, cells were lysed and luciferase activity was measured using the Dual-Luciferase Reporter Assay Kit (Promega).

### Western Blotting

Western blot analysis was performed as described^74^. C11 cells were treated with OTX015 or JQ1 for 24 h and lysed on ice for 30 min. Approximately 50–150 mg of thermally denatured protein extract was loaded on a 10% polyacrylamide gel, electroblotted onto a nitrocellulose membrane and blocked for one hour. The membrane was then incubated with CDK9, Cyclin T1 or CDK9-pT186 antibodies (Cell Signaling Technology). Bands were visualized using the ECL Western blotting system (Santa Cruz Biotechnology).

### Chromatin immunoprecipitation (ChIP)

ChIP assays were performed according to the manufacturer’s protocol (Millipore) and previously described procedures^72^,^75^. Briefly, C11 cells (1 × 10^7^) were treated with OTX015 for six hours, fixed in 1% formaldehyde, resuspended in lysis buffer and sonicated to obtain DNA fragments of 500–1000 bp. DNA fragments were incubated with IgG, CDK9 or Pol II CTD-Ser2P (Abcam) antibodies at 4 °C overnight and immune complexes were retrieved by incubating with Protein G agarose beads. Following washing, the chromatin was eluted and reverse cross-linked overnight. DNA was extracted and quantitative real-time PCR was performed using the SuperReal PreMix Plus (TIANGEN) on a Roche LightCycler 480 II machine with the forward (5′-AGACTGCTGACATCGAGCTTTCT-3′) and reverse primer (5′-GTGGGTTCCCTAGTTAGCCAGAG-3′). Results from fragments obtained after incubation with different antibodies were normalized against input DNA and presented as fold change relative to DMSO control.

### Statistical analysis

Means and standard errors (SE) were calculated for all data points from at least 3 independent experiments in triplicates. Statistical significance was determined using the two-way Student t test, where p value < 0.05 considered significant.

## Additional Information

**How to cite this article**: Lu, P. *et al.* The BET inhibitor OTX015 reactivates latent HIV-1 through P-TEFb. *Sci. Rep.* **6**, 24100; doi: 10.1038/srep24100 (2016).
